# Bachmann's Bundle‐Related Atrial Tachycardias Following Catheter Ablation of Persistent Atrial Fibrillation

**DOI:** 10.1111/jce.70220

**Published:** 2026-01-19

**Authors:** Nate Christian‐Miller, Mohammed Al‐Sadawi, Muazzum Shah, Kelly Arps, Amrish Deshmukh, Jackson J. Liang, Krit Jongnarangsin, Fred Morady, Hakan Oral, Aman Chugh, Michael Ghannam

**Affiliations:** ^1^ Electrophysiology/Division of Cardiology University of Michigan Ann Arbor Michigan USA

**Keywords:** atrial fibrillation, atrial tachycardia, atypical atrial flutter, Bachmann's Bundle, catheter ablation, epicardium, perimitral atrial flutter

## Abstract

**Background:**

Atrial tachycardias (AT) after radiofrequency ablation (RFA) of atrial fibrillation (AF) may utilize Bachmann's bundle (BB). Due to their epicardial location, these ATs remain poorly understood.

**Objective:**

To describe the electrophysiologic and anatomic basis of BB‐related ATs.

**Methods:**

The region of BB was defined as the anterior left atrium (LA) immediately outside the right superior pulmonary vein. A BB‐dependent AT was defined as an arrhythmia that originated from (focal) or involved the BB region (reentrant).

**Results:**

Among 1611 patients with persistent AF undergoing ablation, 32 patients (2%) (age 69 ± 9, male *n* = 22, LA size 47 ± 6 mm, ejection fraction 55 ± 13%) with BB ATs were included. Twenty‐nine (91%) had undergone prior ablation for persistent AF (average, 2.0 ± 1.3 procedures). The mechanism of BB ATs was focal (*n* = 7, 22%) or macro‐reentry (*n* = 25, 78%). RFA eliminated all focal and ultimately reentrant ATs in 15 of the 32 patients; RFA was required at the right atrial (RA) projections of BB among eight of the latter patients. The electrogram at the successful site was devoid of local voltage in four patients. In nine patients with redo procedure, recurrent BB‐AT was found in five (56%). After 2.3 ± 1.4 years of follow‐up, 22 of the 32 patients (69%) remained free of atrial arrhythmias.

**Conclusion:**

The region of the BB bundle may be responsible for focal and reentrant tachycardias following RFA of persistent AF. Given its epicardial location, sequential ablation from the LA and RA may be required, even at sites that might be devoid of local voltage.

## 1 Introduction

Atrial tachycardias (AT) are frequently encountered following catheter ablation of persistent atrial fibrillation (AF). Conventional left atrial (LA) reentrant ATs involving the mitral isthmus and the LA roof are reasonably well described. Not uncommonly, more complex circuits may be encountered that might be difficult to eliminate owing in part to incomplete understanding of the mechanisms. Some of these challenging arrhythmias involve the epicardial atrial myocardium, specifically the region of Bachmann's bundle (BB) [1]. The BB is an epicardial structure located at the interatrial groove. Endocardially, its projections can be localized to the anterior LA, outside the antrum of the right superior pulmonary vein (PV). Due to its epicardial location, mapping and ablation of BB‐related tachycardias may be challenging. The purpose of this study is to describe the anatomic and electrophysiologic characteristics of BB‐related ATs.

## 2 Methods

This is a retrospective study of consecutive patients undergoing ablation of atrial arrhythmias involving BB between 2018 and 2022. The study protocol was approved by the institutional review board of the University of Michigan.

### 2.1 Electrophysiology Study

All patients provided informed written consent. All antiarrhythmic drugs except amiodarone were discontinued at least four to five half‐lives before the study; amiodarone was discontinued at least 2 months before the procedure. Oral anticoagulation was not interrupted. The procedure was performed using general anesthesia. All patients underwent a transesophageal echocardiogram to rule out LA thrombus. After transseptal puncture, systemic anticoagulation was achieved with intravenous heparin to maintain an activated clotting time of > 350 s. A 3‐D mapping system was used to guide catheter navigation and ablation (CARTO 3, Johnson & Johnson MedTech, Irvine, California, USA). An open‐irrigation, contact‐force sensing, 3.5‐mm‐tip deflectable catheter (Thermocool Smarttouch Surroundflow, Johnson & Johnson MedTech) was used for mapping and ablation. Bipolar electrograms were recorded at a band pass of 30–500 Hz (EPMedSystems, West Berlin, NJ). The esophagus was delineated with a radio‐opaque marker. Radiofrequency (RF) energy was applied at a maximum power output of 35 W at a flow rate of 30 mL/min and a maximum temperature of 45°C. When ablation was performed near the PV ostia, or at the posterior LA, the power was reduced to 25 W at a flow rate of 17 mL/min. Power was limited to 20 W during energy application in the coronary sinus (CS).

### 2.2 BB Tachycardia and Mapping of AT

BB tachycadias were identified using previously reported criteria [2, 3] including biatrial tachycardias utilizing epicardal connections, “oblique” perimitral flutters utilizing the lateral LA ridge and BB connections [4], or focal ATs involving BB [5]. Patients in whom activation and pacemapping demonstrated circuits confined to the endocaridum alone were not included.

A high‐density map of the LA was created during AT utilizing a multipolar catheter (Pentaray or Octaray, Johnson & Johnson MedTech). Regions of high real‐time impedance and/or contact force (suggestive of a pouch) encountered during withdrawal (as opposed to advancement) of the ablation catheter were also identified. Entrainment mapping (while pacing at a rate 20 ms shorter than the tachycardia cycle length [CL]) was performed to identify components of the reentry circuit. Sites at which the difference between the return cycle and tachycardia CL was less than or equal to 20 ms were considered to be within the circuit.

A focal mechanism was considered if centrifugal activation from the site of origin could be demonstrated, along with confirmation of presystolic activity (with respect to the surface p‐wave). The definition of macro‐reentry included participation of opposite walls of the atrial chamber, and activation spanning the CL of the tachycardia.

### 2.3 Post‐Ablation Management and Follow‐Up

Patients were monitored on a telemetry unit overnight and prescribed oral anticoagulation and rate‐controlling medications. Antiarrhythmic medications were not prescribed unless the patient developed an acute recurrence associated with hospitalization, severe symptoms, or heart failure. Patients were seen in an outpatient clinic 3 months after the ablation procedure and every 3–6 months thereafter. Rhythm status was assessed via a 30‐day, auto‐trigger event monitor (Lifestar AF Express, Life Watch Inc., Buffalo Grove, IL, USA) or device interrogation in patients with an implantable device, at 12 months after the ablation procedure, in the absence of antiarrhythmic medications. Recurrence was defined as sustained (> 30 s) symptomatic or asymptomatic AF/AT after the 3‐month blanking period.

### 2.4 Statistical Analysis

Continuous variables are expressed as mean ± standard deviation, categorical variables were expressed as counts and percentages. Data were compared using the Fisher's *t* test or *χ* ^2^ test for categorical variables and the Student's *t*‐test or Mann–Whitney test for continuous variables as appropriate. Cox proportional hazard modeling was used to examine patient and procedural characteristics associated with arrhythmia recurrence. A 2‐tailed *p* < 0.05 indicated statistical significance. All statistical analyses were performed using R version 4.1.1 (R Foundation for Statistical Computing, Vienna, Austria).

## 3 Results

Among 1680 patients undergoing ablation for persistent AF, 32 patients (2%) with BB‐related tachycardias were included. The study population included 23 men (72%), with an age of 69 ± 8 years, ejection fraction of 54 ± 12%, and LA diameter of 46 ± 6 mm (Table 1). All patients had a history of persistent AF and had failed antiarrhythmic medications, including six who had failed amiodarone. Twenty‐nine patients (91%) had undergone at least one prior ablation with thermal ablation; the remaining three patients were found to have BB AT during their initial procedure. The average time from the before the current procedure was 1.25 years (range, 0.9–3.3 years). Among patients who underwent a prior ablation procedure, target sites included the PVs (*n* = 29, 100%), the posterior wall (PW) (*n* = 21, 72%), anterior LA (*n* = 10, 34%), septum (*n* = 8, 28%), LA appendage ablation (without isolation) (*n* = 12, 41%), and RA sites (*n* = 15, 52%). Among the three patients undergoing first‐time ablation, after PV and PW isolation, ablation near the LAA (*n* = 2) or the mitral isthmus (*n *= 1) resulted in termination of AF to a BB‐related AT.

**Table 1:** Patient characteristics.

N	32
Age years	69 (8.2)
Males, *n* (%)	23 (72)
BMI	33 (6.4)
EF, %	54 (12)
LA size, mm	46 (6.1)
HTN, *n* (%)	26 (81)
HLD, *n* (%)	18 (56)
DM, *n* (%)	5 (16)
Thyroid disease, *n* (%)	10 (31)
CAD, *n* (%)	7 (22)
PCI, *n* (%)	3 (9)
CABG, *n* (%)	2 (6)
Surgical maze, *n* (%)	2 (6)
SSS, *n* (%)	4 (13)
Valvular heart disease, *n* (%)	2 (6)
OSA, *n* (%)	17 (53)
CVA, *n* (%)	4 (13)
Prior RFA, *n* (%)	
0	3 (9)
1	11 (34)
2	10 (31)
3	6 (19)
4	2 (6)
BB, *n* (%)	28 (88)
CCB, *n* (%)	17 (53)
ACE, *n* (%)	6 (19)
ARB, *n* (%)	10 (31)
ASA, *n* (%)	8 (25)
Statin, *n* (%)	18 (56)
Spironolactone, *n* (%)	4 (13)

*Note:* Values indicate mean and standard deviation unless specified otherwise.

Abbreviations: ACE, angiotensin converting enzyme inhibitors; ARB, angiotensin II receptor blockers; ASA, aspirin; BB, beta blockers; BMI, body mass index; CABG, coronary artery bypass grafting; CAD, coronary artery disease; CCB, calcium channel blockers; CVA, cerebrovascular accident; DM, diabetes mellitus; EF, ejection fraction; HLD, dyslipidemia; HTN, hypertension; LA, left atrium; OSA, obstructive sleep apnea; PCI, percutaneous intervention; RFA, radiofrequency ablation; SSS, sick sinus syndrome.

### 3.1 Procedural Findings

**Figure 1:**
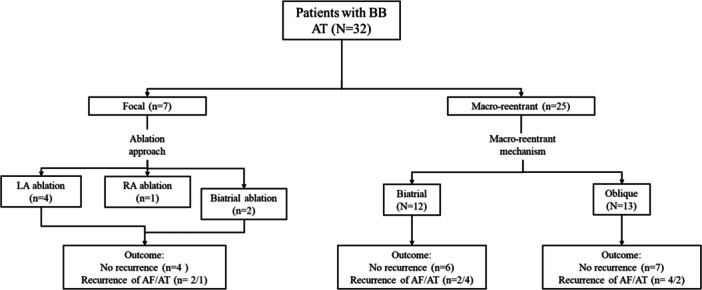
Flowchart showing the course of the study patients.

The presenting rhythm was sinus in 8, AF in 8, or AT in 16 patients. Among the former, arrhythmia induction was performed with rapid atrial pacing alone (*n* = 3) or during isoproterenol infusion (10–20 µg/min; *n* = 5). Among the eight patients presenting in AF, RF ablation terminated AF to an AT involving the BB in six patients (86%) or to sinus rhythm in one patient (14%). One patient presenting in AF underwent extensive ablation followed by transthoracic cardioversion to sinus rhythm, and BB tachycardia was then induced with rapid atrial pacing. Among the six patients in whom RF terminated AF to AT, culprit sites included the base of the LA appendage (*n* = 4), the anterior LA (*n* = 1), and the septal aspect of the base of the RA appendage. Rapid atrial pacing induced a BB‐dependent AT in the one patient who required transthoracic cardioversion after RF ablation of AF (see flowchart in Figure 1).

Among the 16 patients presenting with AT, a BB‐related tachycardia (mean CL, 290 ± 60 ms) was the mechanism in nine patients (56%). The remaining patients presented with perimitral (*n* = 4) or roof‐dependent AT (*n* = 3) with a mean CL of 292 ± 60 ms, which terminated with RF ablation, to a BB AT with a CL of 327 ± 103 ms.

**Figure 2:**
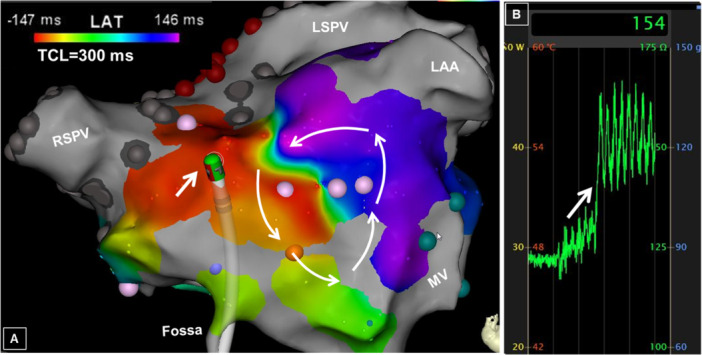
Real‐time impedance and pouch. While mapping an atrial tachycardia (AT) involving the anterior left atrium (LA) with the ablation catheter (arrow, panel A), there is a sudden rise in the real‐time impedance from about 125 ohms to 150 ohms (arrow, panel B), suggestive of a pouch in the region of Bachmann's bundle (BB). Note that mapping accounted for 98% (293/300 ms) of the tachycardia cycle length (TCL), consistent with macro‐reentry. LS, left superior; LAA, left atrial appendage; PV, pulmonary vein; RS, right superior.

**Figure 3:**
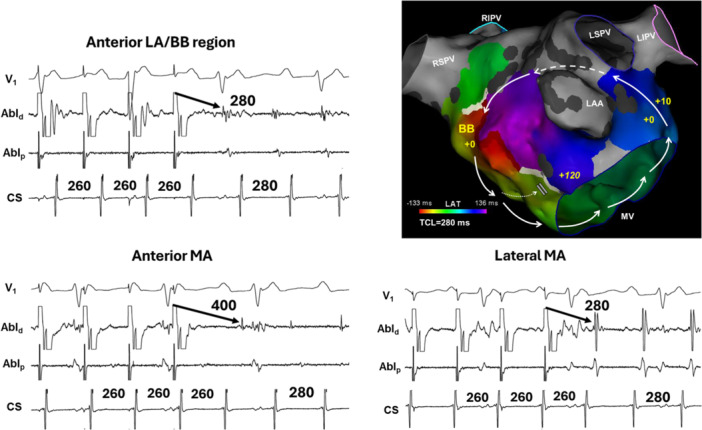
Oblique perimitral reentry. (A) An activation map showing an example of an oblique variant of perimitral atrial flutter in a patient with prior LAA isolation. In this case, the reentrant circuit does not involve the anterior annulus, in contradistinction to classic perimitral reentry, and instead the circuit is completed at the region of BB. The numbers on the map refer to the difference between the post‐pacing interval and the TCL during entrainment mapping. Note the very long return cycle ( + 120 ms) at the anterior annulus (See Figure 2B). RI, right superior; LI, left inferior. (B) Shown are the results of entrainment mapping from the same patient whose activation map is shown in Figure 3A. The post‐pacing interval from the anterior LA (near Bachmann's bundle region) and the lateral mitral isthmus show an in‐circuit response, consistent with macro‐reentry. However, the very long return cycle at the anterior mitral annular region rules out conventional perimitral reentry. Abl, ablation; CS, coronary sinus; MA, mitral annulus.

The mechanism was macro‐reentry in 25 of the 32 patients (78%) with BB‐related tachycardias (Figure 2). Perimitral reentry accounted for the mechanism in 13 of the 25 patients (52%) with an oblique variant diagnosed in all 13 of these patients (Figure 3).

**Figure 4:**
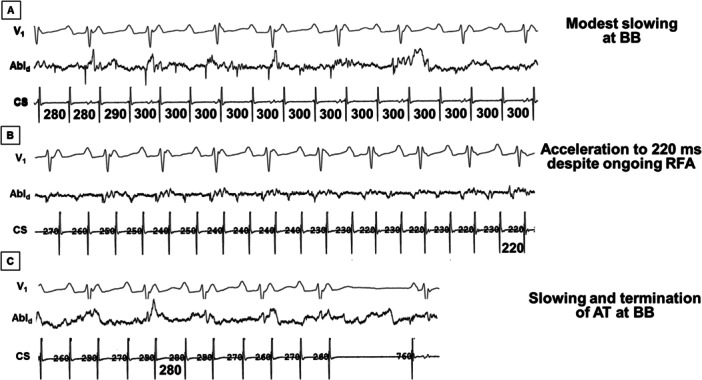
Response to radiofrequency ablation (RFA) ablation at the BB region for oblique perimitral reentry in the same patient whose map and tracings are shown in Figure 3A,B. (A) RF energy delivery slows the AT from 280 ms to 300 ms, but without tachycardia termination. (B) Ongoing energy delivery paradoxically accelerates the tachycardia from 300 ms to 220 ms. (C) Prolonged energy application again slows the tachycardia, finally terminates the AT to sinus rhythm.

**Figure 5:**
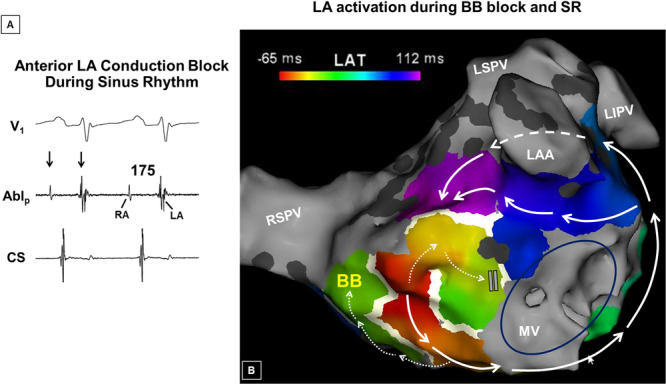
Anterior LA and BB block during sinus rhythm (SR). (A) A widely‐split double potential was recorded at the high anterior wall of the LA from the same patient whose maps and tracings are shown in Figures 3 and 4. The initial smaller electrogram (arrows) represents far‐field right atrial activation (note that it precedes the onset of the p‐wave), and the second signal, local LA activation. (Note that the anterior LA is activated well after the coronary CS). (B) An activation map during SR shows that interatrial activation proceeds via the fossa as opposed to BB, consistent with BB block. As a result, BB is activated in a caudocranial fashion. In the presence of conduction block at the anterior wall as well, high anterior LA (purple region) is activated after the lateral LA. The dashed arrow represents activation along the ridge (between the isolated LAA and the left PVs). Note the LA activation time is very prolonged at 177 ms.

**Figure 6:**
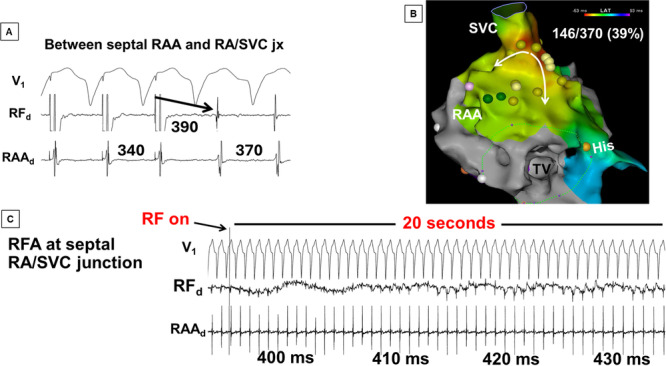
An example of biatrial tachycardia that required ablation at both right (RA) and left atrial aspects of BB. (A) Entrainment mapping from the septal aspect of the base of RA appendage (RAA) shows that this site is a component of the reentrant circuit. (B) An activation map shows that the earliest breakthrough occurred at the septal RA/superior vena cava (SVC) junction, the site corresponding to the right atrial endocardial projections of BB. Note that only a fraction of the tachycardia cycle length (39%) could be accounted for with activation mapping. (C) A prolonged RF energy application at the septal RA/SVC junction (jx) only slows the tachycardia from 400 ms to 430 ms (see next figure).

**Figure 7:**
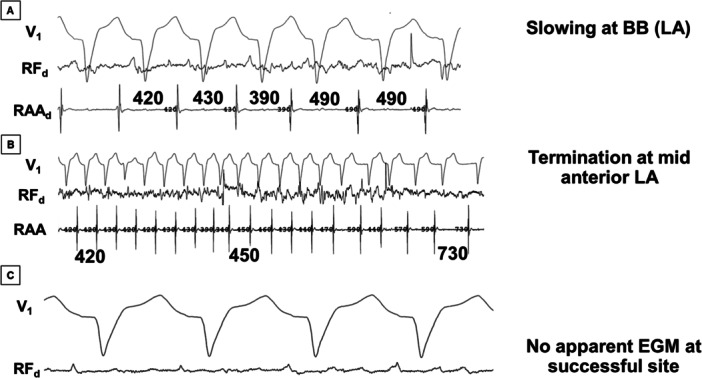
Continued from Figure 6. Slowing and termination of biatrial AT at anterior LA. (A) Since the AT did not terminate during RA ablation, additional ablation was performed at the LA aspect of BB, which further slowed the tachycardia from 420 ms to 490 ms. (B) Additional ablation was required at the high anterior LA for termination despite the absence of a local electrogram (see panel C). (C) Despite high gain setting, no obvious local electrogram is apparent (other than baseline wander/artifact) at the site where RFA terminated the tachycardia to sinus rhythm (see next figure).

**Figure 8:**
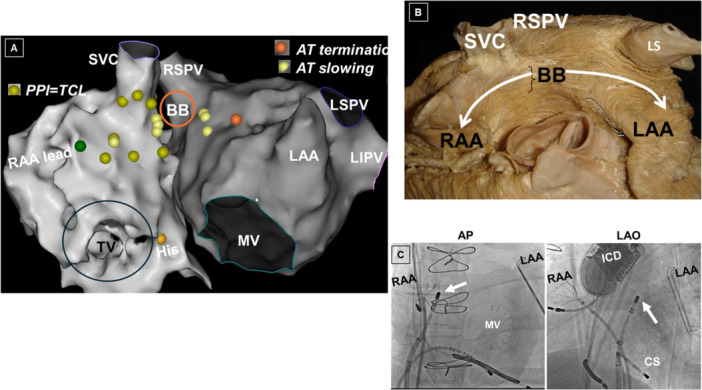
(A) A biatrial map showing components of the reentry circuit (gold tags), sites that slowed AT (yellow tag) and the site that finally terminated AT (orange tag) in the same patient as in Figures 6 and 7. The orange circle represents the epicardial location of Bachmann's bundle. Green tags represent the site of the RA lead. (B) An anatomic specimen showing the relationship of Bachmann's bundle to the LA and the RA. (Modified with permission from Kuhne et al, reference number 12 [6]).(C) Fluoroscopic views showing the location of the ablation catheter in the LA (arrow) where RF ablation terminated the tachycardia. AO, left anterior oblique; AP, anteroposterior; LICD, implantable cardioverter defibrillator generator.

Among patients with macro‐reentrant BB AT, ablation resulted in arrhythmia termination (*n* = 15), slowing without termination (*n* = 8), or no effect (*n* = 2). Acceleration of the tachycardia CL was noted during RF ablation in five patients despite initial slowing (Figure 4). Evidence of BB block was noted in one patient (Figure 5). Ablation at the RA insertion of BB was performed in six patients resulting in arrhythmia termination or slowing in five patients. More extensive linear ablation along the septal base of the RA appendage (anchored to the septal RA/SVC junction, and the anterior tricuspid annulus, as needed) was required in four of nine (44%) patients with refractory biatrial tachycardia (Figures 6, 7, 8). There was no apparent local electrogram, that is, scar, at the site where RF ablation slowed or terminated the tachycardia in four patients (Figure 7).

**Figure 9:**
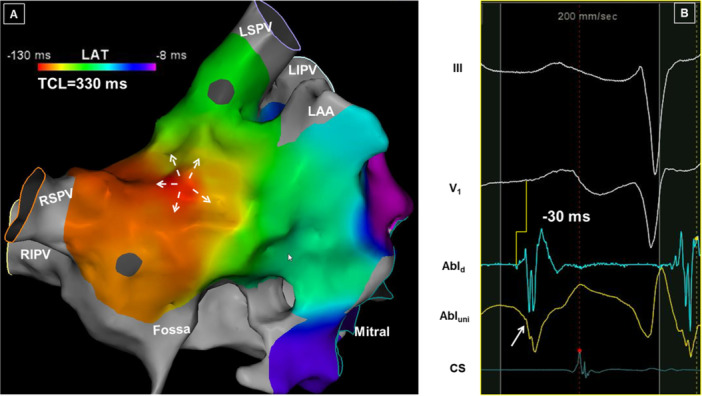
An example of a focal AT from BB region. (A) An activation map shows radial spread from the site of origin at BB. Despite a cycle length of 330 ms, only 122 ms (37%) could be accounted for consistent with a focal mechanism. (B) The electrogram recorded at the site where RFA terminated the tachycardia shows that the local signal precedes the surface p‐wave by 30 ms. Note that a Q‐wave (arrow) is recorded on the unipolar electrogram, again confirming the site of origin.

Focal BB arrhythmias were identified in seven patients (Figure 9). All seven patients had termination of arrhythmias with ablation, including three patients in whom RA ablation was performed to prevent recurrence.

After mapping and ablation of BB‐dependent ATs, re‐isolation (or confirmation) of the PVs and the posterior LA was performed in all patients. Additional ablation was required for inducible AF or AT and included the anterior wall (*n* = 26), mitral isthmus (*n* = 23), and LAA (*n* = 20). The total procedure time for the index ablation was 314 ± 87 min, with a total RF time of 62 ± 16 min, and fluoroscopy time of 29 ± 11 min.

### 3.2 Pouch at BB Region

**Figure 10:**
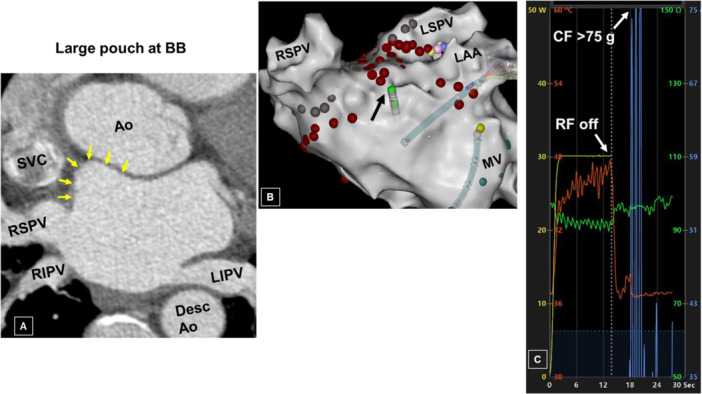
Pouch at the BB region. (A) A computed tomographic axial reconstruction showing a large pouch (arrows) at the region of Bachmann's bundle (BB). Ao, aorta. (B) A 3D anatomic map of the LA during RFA from another patient. As the catheter (black arrow) was deflected anteriorly, it suddenly dislodged, prompting immediate discontinuation of energy delivery (see next). (C) Note the very high contact forces (> 75 g) encountered upon catheter dislodgement into the pouch.

An anatomic pouch was present at the LA site of termination in nine of the 32 patients (28%). Presence of a pouch was heralded by a sudden rise in the real‐time impedance and/or contact force that was not related to advancing the catheter. Before RF ablation in this region, the catheter was gently withdrawn and deflected until these parameters were acceptable. An outpouching at the corresponding region of the anterior LA was present in three of the four (75%) patients who underwent preprocedure CT imaging (Figure 10).

### 3.3 Outcomes

There were no major procedural complications. During a follow‐period of 25 ± 16 months, 15 of the 32 (47%) patients experienced recurrence (AF in 8, and AT in 7). A repeat procedure was performed in nine patients, during which recurrent BB‐related arrhythmia was present in five patients. In four of these patients, the arrhythmia had terminated with ablation at the prior procedure, and had only slowed in the remaining patient. During the repeat procedure in these five patients, BB AT was terminable with ablation in two. The remaining three underwent DCCV at the completion of the procedure, with 1/3 patients experiencing arrhythmia recurrence on follow‐up. Conduction block across the (lateral) mitral isthmus was ultimately obtained in 9/12 (75%) of patients in whom it was attempted. Alcohol ablation of the vein of Marshall was required in three of these patients. The conduction delay during LAA pacing to the distal CS electrogram was 218 ± 68 ms. During a mean follow‐up of 2.3 ± 1.4 years after the last procedure, 22 of the 32 patients (69%) remained arrhythmia‐free with nine patients remaining on antiarrhythmic medications. There were no patient‐ or procedural characteristic predictive of arrhythmia recurrence, including those with focal versus macro‐reentrant tachycardias, and those with slowing versus termination of BB arrhythmias during ablation (*p* > 0.05 for all).

## 4 Discussion

The region of BB may be responsible for maintaining macro‐reentrant and generating focal atrial arrhythmias in patients with persistent AF. Extensive ablation at both the left and right atrial aspects may be required to terminate these arrhythmias. Sites where RF ablation terminated the tachycardia were often devoid of local activity. These findings are consistent with the participation of the epicardial myocardium in the region of the interatrial band. It should then not be surprising that BB‐related AT often recurred during follow‐up, requiring a repeat procedure, even in patients in whom termination was achieved during the prior session. In the absence of direct mapping and ablation of the epicardial myocardium, long‐term success rates after multiple procedures remain modest in patients with BB‐related arrhythmias.

### 4.1 Mechanisms of BB Tachycardias

The mechanism of the BB‐related tachycardias was macro‐reentry in the majority of patients. More specifically, the mechanism in about one‐half of the patients was perimitral reentry, including the oblique variant. As described earlier, the reentry circuit in such cases does not involve the entirety of the mitral annular myocardium, and that the anterior mitral valvular region, in contradistinction to “classic” perimitral reentry, is not part of the reentry circuit. The operator may choose among a number of approaches for perimitral reentry. Either a lateral or an anterior approach (modified in case of oblique reentry) is reasonable. An anterior approach was undertaken in the current series in some patients in hopes of exploiting the presence on anterior wall scarring, specifically, to minimize the extent of linear ablation. In other cases, an anterior approach was required after failure of a lateral approach. The “classic” lateral linear lesion connecting the mitral annulus to the left‐sided PVs remains our preferred initial approach. The advantage of such an approach is that it allows the operator to address the epicardial components, either via the CS (with RF ablation) and/or the vein of Marshall (with chemical ablation). The fact that epicardial components of the reentry circuit cannot be routinely targeted with an anterior approach is a limitation.

Apart from perimitral flutter, the BB region was also implicated in other reentrant tachycardias, spanning from relatively simple circuitry involving the anterior LA (as in Figure 2) to more complex activation patterns. Components of the latter circuits include the left and right atrial projections of BB, and the septal base of the RA appendage, toward the anterior tricuspid annulus. In the absence of dedicated epicardial mapping, it might be difficult to reconstruct the entire circuit of these complex arrhythmias. The role of epicardial circuitry has been elegantly shown in prior reports [7, 8, 9]. Despite epicardial mapping in some cases, RF ablation is often performed endocardially [7]. This observation attests to the challenges in epicardial ablation including the relatively thinness of the LA wall in this region, concerns of arterial injury to the sinus node artery, which might call for antecedent coronary angiography [10], and to BB itself, which may result in interatrial dyssynchrony. Dissociation of the right and left atrium after extensive ablation along the projections of BB alone is unlikely due to intratrial connections via the CS and fossa ovalis [11]; however, this has occurred in patients undergoing extensive ablation procedures [12]. The hemodynamic and long‐term implications of this are not fully understood; a focused approach targeting critical pathways only should be employed when possible.

Apart from reentrant circuits, focal arrhythmias may also arise from the BB region. Unlike the former, which are encountered during the course of extensive ablation for persistent AF, focal BB tachycardias are unlikely to be related to gaps in prior lesions. The BB region is an uncommon source of focal arrhythmias in patients with or without AF [13]. An experimental model showed that unstable reentrant circuits at BB can maintain AF, which can be interrupted with epicardial RF ablation of the bundle [14]. In human AF, which is typified by widespread changes in the atrial substrate, it is unlikely that RF ablation of BB *per se* would be sufficient to eliminate persistent AF.

Patients in this study frequently had extra‐PVI ablation including linear ablation at the anterior wall and septum preceding the development of BB. Post‐ablation AT after persistent AF ablation may be due to a proarrhythmic substrate, particularly when complete linear block cannot be attained [15] and may have contributed to in some patients. Alternatively, given the infrequent nature of these arrhythmias, they may represent direct pathology of BB itself particularly in the case of focal arrhythmias. Further studies are needed to understand patient and procedural risk factors for the development of these arrhythmias.

### 4.2 Anatomic Considerations

In the absence of direct epicardial mapping, several observations provide circumstantial evidence of epicardial involvement. First, tachycardia slowing or termination often occurred at sites without any appreciable local electrograms, even at maximal gain settings. This implies that the salutary effect of ablation was due to heating of relatively remote epicardial components that were critical to the maintenance of tachycardia. RF energy was applied at these sites nonetheless as entrainment mapping at adjacent sites showed an in‐circuit response. Further, the necessity of prolonged (i.e., > 30 s) RF lesions were required before some slowing was noted, and subsequent recovery also implicate the epicardial myocardium.

At first glance, the observation that *right atrial* ablation was required to terminate perimitral reentry in some patients seems difficult to reconcile. During an anterior approach to perimitral reentry, RF ablation may be required at BB if simpler linear lesions (at the high anterior LA) are not effective, especially during the oblique variant. It follows then, if the AT does not terminate after targeting LA projections of BB, energy delivery may be required at the adjacent sites to target the RA projections (near the septal RA/SVC junction). This approach is somewhat analogous to bipolar ablation of a deep septal or intramural substrate maintaining ventricular tachycardia. In the current series, *sequential* ablation of left and then right atrial projections of BB was required to address epicardial circuitry.

Interestingly, acceleration of the tachycardia CL, and then subsequent slowing with additional energy delivery were also noted. Thermal automaticity, exemplified by tachycardia acceleration, is often observed during RF ablation of focal arrhythmias, as well as in the vicinity of the sinoatrial and atrioventricular nodes. The mechanism behind the dramatic acceleration noted during RF ablation of *reentrant* BB‐ATs is unknown. The site of ablation near the right superior PV may colocalize with ganglionic plexi [16]; the impact of neuromodulation on AT termination is not understood and may be the subject of future studies.

### 4.3 BB Pouch

In approximately one‐third of the patients in the study, an outpouching of the anterior LA outside the right superior PV was identified, that is, at the region of BB. These pouches have been previously characterized as diverticula or even accessory LA appendages, and have been identified in about one‐third of patients, with and without a history of AF [17]. “Passive” entry into this structure resulted in a sudden rise in impedance and contact force. In such cases, the catheter must be carefully negotiated out of the pouch before RF application. This maneuver does frequently result in poor contact force as the catheter is withdrawn proximal to the neck of the pouch. Although none of the patients in the current study experienced complications, cardiac perforation has been reported [10] owing to relative thinness of the tissue around the pouch as compared with the adjacent myocardium [11]. A pouch was identified in some patients with pre‐procedural CT imaging, which might facilitate mapping and ablation in this region and help avoid complications.

### 4.4 Clinical Recurrence

Given the epicardial location of BB, it is not entirely surprising that some patients experienced recurrence during follow‐up, despite arrhythmia termination. This observation might be met with a recommendation for higher power ablation in an effort to create deeper lesions. However, such an approach may be associated with an increased risk of collateral injury to the sinus node artery, as has been previously reported [12], and perforation if energy is delivered in the vicinity of a pouch [18]. Epicardial ablation certainly allows the operator to directly target the arrhythmia source but is not routinely performed for atrial arrhythmias even at high‐volume centers. One could also consider empiric LA appendage isolation since this region seems to be a component of complex anterior LA circuits utilizing BB [9]. One must then consider implantation of an occlusion device due to the increased risk of thromboembolism after electrical disconnection of the LA appendage. While not observed in this population, extensive septal ablation may result in dissociation of the right and left atrium. Ablation of BB arrhythmias often requires ablation at epicardial sites both along the length of BB and the lateral LA in the case of biatrial tachycardias utilizing the mitral isthmus. Pulsed field ablation (PFA) for PV isolation has been integrated into routine clinical practice, and smaller series have demonstrated its utility on extra‐PVI ablation as well [19]. The safety, lesion depth, and durability of PFA on epicardial atrial structures remain to be seen [20] and should be the focus of future studies.

## 5 Limitations

This was a single‐center, retrospective study with a small patient population. Epicardial mapping of the BB was not performed, which may have improved understanding of the mechanisms and procedural outcomes. Mapping and ablation from adjacent structures including the pulmonary artery [21] may also be considered. As a retrospective study without a prespecified ablation protocol, a variety of ablation approaches were utilized on initial procedures, including from those referred from outside institutions. All patients underwent ablation with RF energy; we are unable to comment on the use of pulse field ablation and the management of BB arrhythmias.

## 6 Conclusions

BB may be responsible for generating focal and perpetuating reentrant tachycardias following ablation of persistent AF. RF ablation at the region of BB may terminate the tachycardia despite an apparent scar at the critical site, consistent with heating of the epicardium. For challenging cases of perimitral reentry and biatrial tachycardias, RA ablation may be required to target the rightward endocardial projections of BB.

## Funding

The authors received no specific funding for this work.

## Disclosure

The authors have nothing to report.
